# Supplementary material for: Infertility screening in unmarried men: A scoping review protocol
Source: PLoS One. 2026 Feb 18;21(2):e0341470. doi: 10.1371/journal.pone.0341470 (PMC12915929; doi:10.1371/journal.pone.0341470)
Supplement: S2 File — (DOCX) [file pone.0341470.s002.docx]

**S2 File. Search strategy** (Terms used in the search strategy in PubMed database).

| Number | Search terms | | |
| --- | --- | --- | --- |
|  | Concept | | Syntax |
| #1 | **P** | Unmarried men | "Unmarried Men"[tiab] OR “unmarried man”[tiab] OR "Unmarried Male*"[tiab] OR "Single Men"[tiab] OR “single man”[tiab] OR "Single Male*"[tiab] OR "Bachelor Men"[tiab] OR "Bachelor Man"[tiab] OR "Bachelor Male*"[tiab] OR "Never-married Men"[tiab] OR "Never-married Man"[tiab] OR "Never-married Male*"[tiab] OR "Non-married Men"[tiab] OR "Non-married Man"[tiab] OR "Non-married Male*"[tiab] |
| #2 | **C** | Infertility Screening | ((Fertility[tiab] OR "Infertil*"[tiab] OR Sterility[tiab] OR "Subfertil*"[tiab] OR "Sub-fertil*"[tiab] OR "Sub fertil*"[tiab] OR "Male Infertility"[tiab] OR "Male sterility"[tiab] OR "Male Subfertility"[tiab] OR "Male Sub-Fertility"[tiab] OR "Male Sub Fertility"[tiab] OR "Infertile men"[tiab] OR "Primary infertility"[tiab] OR "Secondary infertility"[tiab] OR "Fertility disorder"[tiab] OR "Infecundity"[tiab] OR “Reproductive Health”[tiab] OR “Reproductive Function*”[tiab]) AND (Screening*[tiab] OR “Mass Screening*”[tiab] OR Assessment*[tiab] OR Diagnos*[tiab] OR Evaluation*[tiab] OR Testing[tiab] OR prescreening[tiab])) |
| #3 | 1990/01/01:2025/03/30[dp] | | |

This is preliminary keywords
