# Supplementary material for: Infertility screening in unmarried men: A scoping review protocol
Source: PLoS One. 2026 Feb 18;21(2):e0341470. doi: 10.1371/journal.pone.0341470 (PMC12915929; doi:10.1371/journal.pone.0341470)
Supplement: Attachment — Submitted filename: Response to Reviewers 2025 05 09.docx [file pone.0341470.s004.docx]

Dear Dr. Godwin Banafo Akrong, 2025/05/09

Academic Editor of PLOS ONE

Thank you for your email and the opportunity to revise our manuscript. We appreciate the time and insights of the reviewers and believe that their comments have helped us to significantly improve the manuscript. We have carefully considered all of the comments and have revised the manuscript accordingly.

Below, we provide a point-by-point response to each of the editor and reviewers' comments.

**Journal Requirements:**

Comment 1. Please ensure that your manuscript meets PLOS ONE's style requirements, including those for file naming. The PLOS ONE style templates can be found at <https://journals.plos.org/plosone/s/file?id=wjVg/PLOSOne_formatting_sample_main_body.pdf> and <https://journals.plos.org/plosone/s/file?id=ba62/PLOSOne_formatting_sample_title_authors_affiliations.pdf>.

**Response:** Thank you for this important point. We have carefully reviewed the PLOS ONE style guidelines and made the necessary revisions to ensure compliance. As these revisions primarily involved formatting adjustments, such as file naming and layout, we have not highlighted them. However, we confirm that all changes have been made according to the journal's requirements.

Comment 2. In the online submission form, you indicated that “Data will be available upon reasonable request to the corresponding author.”

All PLOS journals now require all data underlying the findings described in their manuscript to be freely available to other researchers, either 1. In a public repository, 2. Within the manuscript itself, or 3. Uploaded as supplementary information.

This policy applies to all data except where public deposition would breach compliance with the protocol approved by your research ethics board. If your data cannot be made publicly available for ethical or legal reasons (e.g., public availability would compromise patient privacy), please explain your reasons on resubmission and your exemption request will be escalated for approval.

**Response:** No datasets were generated or analysed during the current study. All relevant data from this study will be made available upon study completion. The sentence was corrected.

Comment 3. Please include a separate caption for each figure in your manuscript.

**Response:** We revise the manuscript to include a separate caption for each figure, as requested. (Page 8, Line 183)

**Reviewer #1:**

Thank you for submitting this protocol. This is an interesting subject in our daily fertility care.
I have few comments to address.

Comment 1. The PCCT framework table formatting is confusing in its current form. Please revise for clarity.

**Response:** We have completely reformatted the PCCT table. (Page 5, Line 106)

Comment 2. It is not clear what kind of translation (as mentioned in the acknowledgement) has been conducted using AI.

**Response:** Thank you for raising this point. We revised this section. (Page 12, Lines 255-256)

Comment 3. While it is common practice in a scoping review to skip formal quality appraisal, a brief justification would strengthen the rationale. We have faced this issue before when top tier journals demanded that we assess the quality of included studies in a scoping review.

**Response:** Thank you for your insightful comment regarding the justification for not performing a formal quality appraisal. We appreciate the suggestion to strengthen our rationale. we have revised this section. (Page 11, Lines 222-224)

Comment 4. A final language check would improve readability of the manuscript.

**Response:** Thank you for your suggestion. We perform a language check to improve clarity.

**Reviewer #2:**

The article is written like a proposal. It is futuristic and reads more like a scoping review guideline for the authors, than like a completed research protocol. It is clear on the existing gap in literature about infertility screening in unmarried men and captures what the authors research intents are, including the protocol they plan to adopt. As a guideline for embarking on the scoping review, it offers sufficient detail on the processes and procedures it plans to follow. However, it is lacking in detail as to whether these activities have actually been undertaken, how they were undertaken, the processes for administering or applying the PRISMA-ScR protocol, what the outcomes are and the implications for future research in that field.

As it stands, the scoping review is yet to be conducted.

**Response:**. Thank you for your precise feedback. As our title "A Scoping Review Protocol" indicates, this manuscript is indeed a protocol, not a completed study. Therefore, the details on execution and outcomes, which you correctly noted are absent, are not yet available as the review is yet to be conducted. Our aim was to transparently present a detailed research plan prior to its execution.

**Other Observations:**

Comment 1. In line 65, mention is made of polycystic ovary syndrome, this is not a factor in male infertility.

**Response:**. Thank you for your careful review and for identifying this error. You are absolutely correct; Polycystic Ovary Syndrome (PCOS) is related to female infertility and is not a factor in male infertility, which is the topic of our study. We apologize for this oversight. We have removed the mention of Polycystic Ovary Syndrome in the revised manuscript to ensure the text accurately reflects factors relevant to male infertility.

Comment 2. In line 173, 'we've' should be written in full as 'we have'.

**Response:** Thank you for this comment. We agree that formal academic writing standards should be maintained. As suggested, we have changed 'we've' to 'we have' in the revised manuscript. We have also checked the document.

While revising your submission, please upload your figure files to the Preflight Analysis and Conversion Engine (PACE) digital diagnostic tool, [https://pacev2.apexcovantage.com/](https://track.editorialmanager.com/CL0/https:%2F%2Fpacev2.apexcovantage.com%2F/1/010f019595c7946a-d7948cc1-9fdf-439c-8b44-e645c1f57b77-000000/GfdVlmzbVdNsNpJAAcKcdoxNqDnHRCpiiz-0WQbuNJQ=202). PACE helps ensure that figures meet PLOS requirements. To use PACE, you must first register as a user. Registration is free. Then, login and navigate to the UPLOAD tab, where you will find detailed instructions on how to use the tool. If you encounter any issues or have any questions when using PACE, please email PLOS at [figures@plos.org](mailto:figures@plos.org). Please note that Supporting Information files do not need this step.

**Response:** We add a figure in this manuscript with requirements checking.
