# Supplementary material for: Infertility screening in unmarried men: A scoping review protocol
Source: PLoS One. 2026 Feb 18;21(2):e0341470. doi: 10.1371/journal.pone.0341470 (PMC12915929; doi:10.1371/journal.pone.0341470)
Supplement: Attachment — Submitted filename: Response to Reviewers 2025 08 25.docx [file pone.0341470.s006.docx]

Dear Dr. Godwin Banafo Akrong, 2025/08/25

Academic Editor of PLOS ONE

Thank you for your email and the opportunity to revise our manuscript. We appreciate the time and insights of the reviewers and believe that their comments have helped us to significantly improve the manuscript. We have carefully considered all of the comments and have revised the manuscript accordingly.

Below, we provide a point-by-point response to each of the editor and reviewers' comments.

Comment 1. The methods should be detailed enough to ensure reproducibility and prevent undisclosed flexibility.

**Response:** We thank the reviewer for emphasizing the critical importance of reproducibility and methodological transparency. Guided by this principle, and incorporating the other specific suggestions from the reviewers, we have revised the Methods section to enhance its level of detail and clarity.

Comment 2. Include appropriate controls, sample size calculations, and replication to ensure robust and reproducible data.

**Response:** We thank the reviewer for their feedback. We wish to clarify that as a protocol for a scoping review, our methodology is based on knowledge synthesis. Therefore, elements of primary experimental studies such as 'control groups' and 'sample size calculations' are not applicable. Robustness is ensured through a detailed and transparent method designed for reproducibility, as is standard for this type of review.

Comment 3. The exploratory aspects of the analysis should be explicitly described.

**Response:** We thank the reviewer for this valuable suggestion. We have revised the Methods section to explicitly describe the exploratory nature of our analysis. We have clarified that we will use an inductive thematic analysis to allow themes and patterns to emerge directly from the data, which aligns with the exploratory goals of a scoping review.

Comment 4. Reference was made to the PRISMA-ScR framework, but the protocol does not fully adhere to its 14-step process.

**Response:** We thank the reviewer for highlighting the need for this important clarification. We wish to explain the distinct roles of the two frameworks mentioned in our manuscript. The 14-step process is the methodology we will follow to conduct the review. This protocol adheres to that process by prospectively detailing how each of the 14 steps will be executed. The PRISMA-ScR, on the other hand, is the guideline we will use for reporting the findings in the final, completed manuscript. We believe this distinction resolves the perceived discrepancy. We have also revised the manuscript to state this separation more explicitly to prevent confusion for readers.

Comment 5. Specify whether data synthesis will be conducted inductively, deductively, or both.

**Response:** We thank the reviewer for this question regarding our synthesis method. Our data synthesis will be primarily inductive. We have clarified in the manuscript that we will use thematic analysis to allow themes and patterns to emerge directly from the data, consistent with the exploratory nature of a scoping review.

Comment 6. The exclusion criteria and data synthesis process require further refinement and clarification.

**Response:** We thank the reviewer for this summary feedback. In line with the reviewer's specific comments, we have thoroughly revised both the exclusion criteria and the data synthesis process. We have added significant clarification to both sections to ensure they are methodologically robust and transparent, and we believe our revisions fully address the reviewer's concerns.

**Journal Requirements:**

Comment 1. If the reviewer comments include a recommendation to cite specific previously published works, please review and evaluate these publications to determine whether they are relevant and should be cited. There is no requirement to cite these works unless the editor has indicated otherwise.

**Response:** We thank the editor for the clear guidance on handling reviewer-suggested citations. We confirm we have followed this principle throughout our revision process.

**Reviewer #1:**

Comment 1. The authors indicate that their submission is a scoping review protocol designed to transparently present a detailed research plan prior to its execution, not the actual completed review. The article offers comprehensive insights into what the authors intend to do, and evidences an in-depth review of literature on the subject matter.

However, I doubt that the use of the word 'protocol' to describe the article is appropriate, particularly as they allude to the use of the PRISMA-ScR which is a known, widely used scoping review protocol. Consequently, referring to their submission as a protocol may only confuse the intended audience, since this submission actually reads like a research design/methodology that is focused on a specific area of research.

**Response:** We thank the reviewer for their valuable feedback on our use of the term 'protocol' and for prompting an important clarification. We have used the term 'protocol' as the manuscript outlines our a priori study plan, which aligns with the standard definition in evidence synthesis. The PRISMA-ScR, in contrast, is the reporting guideline we will follow for the completed review, not the protocol for its execution. To ensure this distinction is perfectly clear to all readers, we have identified the sentence that may have caused ambiguity and have revised it in the manuscript. The sentence now explicitly states: "The completed scoping review will be reported in accordance with the PRISMA-ScR guidelines." (Page 2, Line 3/ Page 6, Line 115). By making this revision, we believe any potential confusion has been removed. Therefore, we have retained the term 'protocol' as it most accurately describes the nature of this manuscript. We are grateful to the reviewer for helping us improve the clarity of our text.

Comment 2. They state in lines 16 and 17 that 'the aim of this scoping review is to understand the scope and nature of evidence related to infertility screening in unmarried men'; and in lines 37 to 39, that 'this scoping review will summarize the evidence on infertility screening in unmarried men, highlighting diverse types of screening, potential benefits and harms/drawbacks of infertility screening, and knowledge gaps.' These statements seem to imply that the article is a scoping review report, yet it only reflects some, not all of the 14-step framework outlined in lines 124 to 137, and so is inconclusive.

On this basis, I advise that the scoping review be conducted and properly reported, such that it effectively captures all the stages of the 14-step framework, and the primary and secondary outcomes outlined in the objective. This will prevent any lack of clarity or confusion on the actual focus of the article.

Thereafter, it can be resubmitted for consideration for publication.

**Response:** We thank the reviewer for their time and detailed feedback. We believe there may have been a misunderstanding regarding the nature of our manuscript, and we appreciate the opportunity to clarify its scope and purpose. This manuscript is a scoping review protocol, not the report of a completed review. Its specific purpose is to outline the detailed methodological plan before the review is conducted. This practice of publishing protocols is standard in evidence synthesis to ensure transparency and methodological rigor. The reviewer correctly observes that our manuscript describes the aims of the future review (lines 16-17 and 37-39) and details a 14-step framework without reporting on the completion of all steps. This is by design. A protocol is intended to present the plan—covering the initial stages of the framework (e.g., defining the question, criteria, and search strategy) and describing how the subsequent stages (e.g., data charting, analysis, and reporting) will be executed. The current manuscript serves precisely this role. Regarding the reviewer's recommendation to conduct the review and resubmit the full report: we agree this is the essential next phase of our research. The completed scoping review will be written up as a separate manuscript for publication once it is finished. However, the explicit goal of this current submission is to have the methodology peer-reviewed and published as a standalone protocol beforehand. To prevent this confusion for future readers, we have carefully reviewed our abstract and introduction to ensure the term "protocol" is stated prominently and the future tense is used consistently.

We hope this clarifies that our manuscript should be evaluated as a study protocol, not a completed review. We believe that when viewed as a research plan, it is comprehensive and conclusive in its own right.

**Reviewer #2:**

Thank you for the opportunity to review this manuscript. The authors planned to review the scope of the literature on screening for male infertility among unmarried men. This is a usual research piece.

Find my few comments below:

**Abstract:**

Comment 1. Structure the abstract as follows: Background/Introduction including the primary goal, Methods including the eligibility criteria, Results (not applicable), Conclusion (not applicable). The protocol should focus on the background, objective and methods.

**Response:** We thank the reviewer for this constructive suggestion. We agree that this structure improves clarity and immediately identifies the manuscript as a protocol. The abstract has been revised accordingly to include the headings: Introduction, Methods, Results (not applicable), Conclusion (not applicable), and Discussion. (Pages 1-2, Lines 14-37)

**Methods**

Comment 2. This is a study protocol, kindly use future tenses not past tense. See lines, 112, 113, 140, 154, 158, 165, 166, 175, 176 and so on. Replace "was, were" with "will" as appropriate.

**Response:** We thank the reviewer for this important correction. The reviewer is correct; as a protocol, the manuscript should be in the future tense. We have now revised the entire document and changed all past tenses to the future tense as requested.

Comment 3. In the exclusion criteria, remove the exclusion of married men. This is because the study included only unmarried men, hence, by definition married men are excluded.

**Response:** We thank the reviewer for this logical correction. The redundant exclusion criterion for "married men" has been removed from the manuscript as suggested. (Page 5, Line 102)

Comment 4. Consider including only original articles with primary or may be secondary data. I don't think review articles should be included.

**Response:** We thank the reviewer for this valuable methodological suggestion. We agree, and have revised our eligibility criteria to include only original studies (with primary or secondary data) and to explicitly exclude review articles. (Page 5 102, Line)

Comment 5. Exclusion of articles should be only based on not meeting the exclusion criteria, not necessarily those who the index authors could get the full article from the authors. However, if an article is considered to be included but the authors could not get access to the full manuscript, a consideration could be made to extract information from the abstract or consider these set of studies as a possible limitation for the scoping review.

**Response:** We thank the reviewer for this crucial methodological point. We have clarified in our protocol that articles will not be excluded based on the unavailability of their full text. Instead, the number of any potentially eligible but irretrievable articles will be reported and discussed as a limitation of the study. (Page 8, Lines 170-172)

Comment 6. The authors categorized the selected articles as included, probably included and excluded. How will the "probably included" be finally disposed?

**Response:** We thank the reviewer for this request for clarification. The "probably included" category is a temporary designation during the initial title/abstract screening. All these articles will undergo a full-text review, after which a definitive 'include' or 'exclude' decision will be made based on our eligibility criteria. (Page 8, Line 169)

Comment 7. The process of data synthesis: Is this going to be done inductively or deductively or both?

**Response:** We thank the reviewer for this insightful question. Our data synthesis will be primarily inductive. We will use thematic analysis to allow themes and patterns to emerge directly from the charted data, which is consistent with the exploratory nature of a scoping review. We have clarified this in the manuscript. (Page 11, Lines 223-225)

While revising your submission, please upload your figure files to the Preflight Analysis and Conversion Engine (PACE) digital diagnostic tool, [https://pacev2.apexcovantage.com/](https://track.editorialmanager.com/CL0/https:%2F%2Fpacev2.apexcovantage.com%2F/1/010f019595c7946a-d7948cc1-9fdf-439c-8b44-e645c1f57b77-000000/GfdVlmzbVdNsNpJAAcKcdoxNqDnHRCpiiz-0WQbuNJQ=202). PACE helps ensure that figures meet PLOS requirements. To use PACE, you must first register as a user. Registration is free. Then, login and navigate to the UPLOAD tab, where you will find detailed instructions on how to use the tool. If you encounter any issues or have any questions when using PACE, please email PLOS at [figures@plos.org](mailto:figures@plos.org). Please note that Supporting Information files do not need this step.

**Response:** We add a figure in this manuscript with requirements checking.
