# Supplementary material for: Infertility screening in unmarried men: A scoping review protocol
Source: PLoS One. 2026 Feb 18;21(2):e0341470. doi: 10.1371/journal.pone.0341470 (PMC12915929; doi:10.1371/journal.pone.0341470)
Supplement: Attachment — Submitted filename: Comments2.docx [file pone.0341470.s005.docx]

**Comment 1**

Abstract: Please remove results (not applicable), Conclusion (not applicable), just don’t include the heading in the abstract. Just write the Introduction, Methods, and go straight to Discussion.

**Comment 2**

PCC line 102 under participants: Correct grammar in this; **Unmarried men with receiving infertility treatment or married women. What does that mean?**

**Comment 3**

Excludes men "receiving infertility treatment" please clarify

**Comment 4**

Write this in full; Infertility Screening (methods, protocols, practices, …), this is so unclear/ vague, what specific aspects of screening.

It is important to define this term for clarity: infertility screening – in this protocol what are you referring to? Clearly define the terms screening and treatment such that they are distinguished. What methods are you referring to?

**Comment 5**

**Context – This is too vague, a**ny geographical location and setting, with no exclusion seems very broad appears unrealistically. What are your intentions? Limiting to certain healthcare settings?

1. Are the authors truly interested in ALL contexts equally?
2. How will they synthesize findings across vastly different settings?
3. Will contextual variation be explored as part of the analysis?

**Comment 6**

After reading this protocol again, I still have so many queries:

While the authors mention "evolving societal norms" and "changing relationship patterns" (lines 48-49), the rationale for specifically focusing on unmarried men rather than all men has not been fully justified.

1. Why does marital status matter for screening approaches, protocols, or outcomes?
2. How do screening considerations differ between married and unmarried men beyond the absence of a partner?
3. What unique barriers, facilitators, or clinical considerations apply specifically to unmarried men?
4. What is driving this focus on unmarried men driven? Is it clinical or something else? Please make it clear and understandable

**Comment 7**

Perhaps the authors should include a **Definitions and Scope** section that clearly delineates all ambiguous terms. A protocol is supposed to be reproducible, and this can only be achieved if all sections are clear and well defined. This protocol needs additional work to enhance its reproducibility; clarity is crucial.
