# Supplementary material for: Infertility screening in unmarried men: A scoping review protocol
Source: PLoS One. 2026 Feb 18;21(2):e0341470. doi: 10.1371/journal.pone.0341470 (PMC12915929; doi:10.1371/journal.pone.0341470)
Supplement: Attachment — Submitted filename: Response to Reviewers 2025 10 23.docx [file pone.0341470.s007.docx]

Dear Dr. Godwin Banafo Akrong, 2025/10/23

Academic Editor of PLOS ONE

Thank you for your email and the opportunity to revise our manuscript. We appreciate the time and insights of the reviewers and believe that their comments have helped us to significantly improve the manuscript. We have carefully considered all of the comments and have revised the manuscript accordingly.

Below, we provide a point-by-point response to each of the editor and reviewers' comments.

**Journal Requirements:**

Comment 1. If the reviewer comments include a recommendation to cite specific previously published works, please review and evaluate these publications to determine whether they are relevant and should be cited. There is no requirement to cite these works unless the editor has indicated otherwise.

**Response:** Thank you for this clarification.

Comment 2. Please review your reference list to ensure that it is complete and correct. If you have cited papers that have been retracted, please include the rationale for doing so in the manuscript text, or remove these references and replace them with relevant current references. Any changes to the reference list should be mentioned in the rebuttal letter that accompanies your revised manuscript. If you need to cite a retracted article, indicate the article’s retracted status in the References list and also include a citation and full reference for the retraction notice.

**Response:** Thank you for the detailed instructions. We have reviewed our entire reference list for accuracy, completeness, and correct formatting. We utilized Zotero to specifically check for any retracted articles, and we confirm that none were found in our references.

**Reviewer #4:**

Comment 1. Abstract: Please remove results (not applicable), Conclusion (not applicable), just don’t include the heading in the abstract. Just write the Introduction, Methods, and go straight to Discussion.

**Response:** Thank you for this clarification. We have revised the abstract structure as requested.

Comment 2. PCC line 102 under participants: Correct grammar in this; Unmarried men with receiving infertility treatment or married women. What does that mean?

**Response:** Thank you for identifying this error. We apologize for the severe confusion. The line you noted (Unmarried men... or married women) mistakenly listed two of our Exclusion Criteria under the 'Participants' section. We have now revised this. (Pages 5/Lines 110)

Comment 3. Excludes men "receiving infertility treatment" please clarify.

**Response:** Thank you. We excluded men 'receiving infertility treatment' because our study focuses specifically on infertility screening. Men who are already in treatment have already passed the screening and diagnosis phase. They represent a 'treatment population,' not a 'screening population,' and including them would confuse these two distinct stages of care. We have clarified this rationale in the manuscript. (Pages 5-6/Lines 110-115)

Comment 4. Write this in full; Infertility Screening (methods, protocols, practices, …), this is so unclear/ vague, what specific aspects of screening.
           It is important to define this term for clarity: infertility screening – in this protocol what are you referring to? Clearly define the terms screening and treatment such that they are distinguished. What methods are you referring to?

**Response:** Thank you for this crucial feedback. You are absolutely correct that our initial description 'Infertility Screening (methods, protocols, practices, …)' was unacceptably vague and failed to provide necessary clarity. We sincerely apologize for this major omission. To address this, we have substantially revised the manuscript to include precise operational definitions. (Pages 5-6/Lines 110-115)

Comment 5. Context – This is too vague, any geographical location and setting, with no exclusion seems very broad appears unrealistically. What are your intentions? Limiting to certain healthcare settings?

1. Are the authors truly interested in ALL contexts equally?

2. How will they synthesize findings across vastly different settings?

3. Will contextual variation be explored as part of the analysis?

**Response:** Thank you for this valid point. We acknowledge that 'any context' appears unrealistically broad. As this is a scoping review, this breadth is intentional. Our primary goal is not to synthesize results, but to map the existing evidence and identify gaps. A central part of our analysis is to extract 'Context' (e.g., country, setting) and descriptively analyze this variation. We have revised the 'Context' section in the manuscript to clarify that our aim is to map and descriptively analyze this contextual variation, not to conduct a quantitative synthesis, which would be unrealistic. (Pages 5-6/Lines 110-115)

Comment 6. After reading this protocol again, I still have so many queries:
           While the authors mention "evolving societal norms" and "changing relationship patterns" (lines 48-49), the rationale for specifically focusing on unmarried men rather than all men has not been fully justified.

1. Why does marital status matter for screening approaches, protocols, or outcomes?

2. How do screening considerations differ between married and unmarried men beyond the absence of a partner?

3. What unique barriers, facilitators, or clinical considerations apply specifically to unmarried men?

4. What is driving this focus on unmarried men driven? Is it clinical or something else? Please make it clear and understandable.

**Response:** Thank you for this critical and insightful feedback. You are absolutely correct that our initial rationale was weak and failed to adequately justify the central premise of our study. We sincerely apologize for this major lack of clarity and have extensively revised the Introduction to make this justification clear and explicit. (Pages 2-3/Lines 45-56)

Comment 7. Perhaps the authors should include a Definitions and Scope section that clearly delineates all ambiguous terms. A protocol is supposed to be reproducible, and this can only be achieved if all sections are clear and well defined. This protocol needs additional work to enhance its reproducibility; clarity is crucial.

**Response:** Thank you for this excellent and highly constructive suggestion. You are absolutely correct that the protocol's reproducibility was weakened by several ambiguous terms, and we sincerely apologize for this. We have fully adopted your recommendation to improve clarity. We have now added a new dedicated subsection in our Methods section titled Key Definitions and Scope. (Pages 7-8/Lines 145-164)
