# Supplementary material for: Infertility screening in unmarried men: A scoping review protocol
Source: PLoS One. 2026 Feb 18;21(2):e0341470. doi: 10.1371/journal.pone.0341470 (PMC12915929; doi:10.1371/journal.pone.0341470)
Supplement: Attachment — Submitted filename: Response to Reviewers 2025 11 17.docx [file pone.0341470.s009.docx]

Dear Dr. Godwin Banafo Akrong, 2025/11/17

Academic Editor of PLOS ONE

Thank you for your email and the opportunity to revise our manuscript. We appreciate the time and insights of the reviewers and believe that their comments have helped us to significantly improve the manuscript. We have carefully considered all of the comments and have revised the manuscript accordingly.

Below, we provide a point-by-point response to each of the editor and reviewers' comments.

**Journal Requirements:**

Comment 1. If the reviewer comments include a recommendation to cite specific previously published works, please review and evaluate these publications to determine whether they are relevant and should be cited. There is no requirement to cite these works unless the editor has indicated otherwise.

**Response:** Thank you for this clarification.

Comment 2. Please review your reference list to ensure that it is complete and correct. If you have cited papers that have been retracted, please include the rationale for doing so in the manuscript text, or remove these references and replace them with relevant current references. Any changes to the reference list should be mentioned in the rebuttal letter that accompanies your revised manuscript. If you need to cite a retracted article, indicate the article’s retracted status in the References list and also include a citation and full reference for the retraction notice.

**Response:** Thank you for the detailed instructions. We have reviewed our entire reference list for accuracy, completeness, and correct formatting. We utilized Zotero to specifically check for any retracted articles, and we confirm that none were found in our references.

**Reviewer #3:**

Thank you for the opportunity to review the revised manuscript. The authors have responded satisfactorily to the initial comments. However, a few inconsistencies as shown below:

Comment 1. In the abstract section, review articles were included while in the method section (i.e., eligibility), reviews were excluded. I suggest you exclude review articles from the scoping review. If they are previous reviews on the subject, you will need to justify why the current review.

**Response:** We appreciate you pointing out this discrepancy. We apologize for the confusion caused by the error in the abstract. We agree with your suggestion and confirm that review articles are excluded from this scoping review. We have revised the Abstract section to ensure it is fully consistent with the eligibility criteria outlined in the Methods section. (Page 2/Line 28)

Comment 2. Kindly expunge unmarried women from the exclusion criteria. The review focuses on unmarried man. There is no need excluding the unmarried women because by the including only unmarried men, the unmarried women are already excluded.

**Response:** Thank you for this precise observation. We have removed "unmarried women" from the exclusion criteria section. (Page 5/Line 110)

Comment 3. How many members of the team will be involved in reviewing the selection in Raygan?

**Response:** Thank you for seeking clarification on this point. We have revised the Data Management and Selection process section to explicitly state that two independent reviewers will be involved in the screening process within Rayyan software. (Page 9/Lines 194-201)

**Reviewer #4:**

Comment 1. Although the response to my Comment 4 was not entirely satisfactory, I noted that the authors addressed this point in the Key Definitions and Scope section. Overall, the authors have adequately addressed my queries, and I believe the manuscript provides valuable knowledge and is suitable for publication.

**Response:** We would like to express our sincere gratitude for your time and meticulous review of our manuscript. We are pleased to hear that the revisions made in the "Key Definitions and Scope" section satisfactorily addressed your concerns regarding Comment 4. We truly appreciate your positive evaluation and recommendation for publication.
