# Supplementary material for: Infertility screening in unmarried men: A scoping review protocol
Source: PLoS One. 2026 Feb 18;21(2):e0341470. doi: 10.1371/journal.pone.0341470 (PMC12915929; doi:10.1371/journal.pone.0341470)
Supplement: Attachment — Submitted filename: PLOS ONE review December 2025.docx [file pone.0341470.s008.docx]

PLOS ONE

**Infertility screening in unmarried men: A scoping review protocol**

The manuscript reports a scoping review protocol designed to understand the scope and nature of evidence related to infertility screening in unmarried men. This is an important issue in reproductive health and has had limited investigation globally. The protocol is detailed and is well written, and I believe would lead to a more substantive understanding of the research question in the international literature.

I have only a few recommendations as follows:

*Novelty* – the research question is novel and will galvanize future research, policy, and practice around infertility in unmarried men.

*Internal validity* – the protocol for the review is well described and if well implemented will enable replicability and the elicitation of accurate research results. However, the question of men who may not be married but may have experienced previous fertility or attempts at fertility was not addressed. This should be considered either as an exclusion criterion, or possibly as a limitation of the study.

*External validity* – I believe the study strengths and limitations as well as the implications of the results for policy and practice should be discussed under the section on discussion.

*Grammatical flow and understanding* – the manuscript reads well with good flow of ideas. However, there are a few typos that need to be addressed before the paper is accepted.

My recommendation is minor revision and resubmission.
