# Supplementary material for: Infertility screening in unmarried men: A scoping review protocol
Source: PLoS One. 2026 Feb 18;21(2):e0341470. doi: 10.1371/journal.pone.0341470 (PMC12915929; doi:10.1371/journal.pone.0341470)
Supplement: Attachment — Submitted filename: Response to Reviewers 2025 12 19.docx [file pone.0341470.s010.docx]

Dear Dr. Godwin Banafo Akrong, 2025/12/19

Academic Editor of PLOS ONE

Thank you for your email and the opportunity to revise our manuscript. We appreciate the time and insights of the reviewers and believe that their comments have helped us to significantly improve the manuscript. We have carefully considered all of the comments and have revised the manuscript accordingly.

Below, we provide a point-by-point response to each of the editor and reviewers' comments.

**Journal Requirements:**

Comment 1. If the reviewer comments include a recommendation to cite specific previously published works, please review and evaluate these publications to determine whether they are relevant and should be cited. There is no requirement to cite these works unless the editor has indicated otherwise.

**Response:** Thank you for this clarification.

Comment 2. Please review your reference list to ensure that it is complete and correct. If you have cited papers that have been retracted, please include the rationale for doing so in the manuscript text, or remove these references and replace them with relevant current references. Any changes to the reference list should be mentioned in the rebuttal letter that accompanies your revised manuscript. If you need to cite a retracted article, indicate the article’s retracted status in the References list and also include a citation and full reference for the retraction notice.

**Response:** Thank you for the detailed instructions. We have reviewed our entire reference list for accuracy, completeness, and correct formatting. We utilized Zotero to specifically check for any retracted articles, and we confirm that none were found in our references.

**Reviewer #4:**

Comment 1. Dear Authors,

Thank you for your thorough revisions. You have addressed all my comments clearly and comprehensively. The manuscript is now much stronger, and I have no further concerns. Well done.

**Response:** We would like to express our sincere gratitude for your review and encouraging comments. We are glad that our revisions addressed your concerns effectively.

**Reviewer #5:**

The manuscript reports a scoping review protocol designed to understand the scope and nature of evidence related to infertility screening in unmarried men. This is an important issue in reproductive health and has had limited investigation globally. The protocol is detailed and is well written, and I believe would lead to a more substantive understanding of the research question in the international literature.
I have only a few recommendations as follows:

Comment 1. Novelty

– the research question is novel and will galvanize future research, policy, and practice
around infertility in unmarried men.

**Response:** We would like to thank the reviewer for recognizing the novelty and significance of this research question. We are grateful for your positive evaluation.

Comment 2. Internal validity

– the protocol for the review is well described and if well implemented will enable replicability and the elicitation of accurate research results. However, the question of men who may not be married but may have experienced previous fertility or attempts at fertility was not addressed. This should be considered either as an exclusion criterion, or possibly as a limitation of the study.

**Response:** Thank you for raising this critical point regarding internal validity. We agree that including men with a history of fertility issues or previous attempts at conception could bias the results, as our focus is on screening. Therefore, based on your recommendation, we have added "Men with a history of previous fertility or attempts at conception" to the exclusion criteria to ensure the study population is clearly defined. (Page 5/Line 110; page 7/Line 153-154)

Comment 3. External validity

– I believe the study strengths and limitations as well as the implications of the results for policy and practice should be discussed under the section on discussion.

**Response:** We appreciate the reviewer’s recommendation. Accordingly, we have ensured that the Discussion section now covers the study’s strengths, limitations, and the potential implications for policy and practice, as suggested. (Pages 12-13/Lines 262-281)

Comment 4. Grammatical flow and understanding

– the manuscript reads well with good flow of ideas. However, there are a few typos that need to be addressed before the paper is accepted.

**Response:** Thank you for your positive feedback on the manuscript's flow. We apologize for the typographical errors. We have carefully proofread the entire manuscript again and corrected all identified typos and grammatical slips to ensure the text is error-free.
